# Interleukin-17 Links Inflammatory Cross-Talks Between Comorbid Psoriasis and Atherosclerosis

**DOI:** 10.3389/fimmu.2022.835671

**Published:** 2022-04-13

**Authors:** Yan Wang, Jinxin Zang, Chen Liu, Zhongrui Yan, Dongmei Shi

**Affiliations:** ^1^ College of Clinical Medicine, Jining Medical University, Jining, China; ^2^ Department of Neurology, Jining No.1 People’s Hospital, Jining, China; ^3^ Laboratory of Medical Mycology, Jining No.1 People’s Hospital, Jining, China; ^4^ Department of Dermatology, Jining No.1 People’s Hospital, Jining, China

**Keywords:** interleukin (IL)-17, interleukin (IL)-17A, IL-23 / IL-17 axis, cardiovascular disease, atherosclerosis, psoriasis

## Abstract

Psoriasis is a chronic, systemic, immune-mediated inflammatory disorder that is associated with a significantly increased risk of cardiovascular disease (CVD). Studies have shown that psoriasis often coexists with atherosclerosis, a chronic inflammatory disease of large and medium-sized arteries, which is a major cause of CVD. Although the molecular mechanisms underlying this comorbidity are not fully understood, clinical studies have shown that when interleukin (IL)-17A inhibitors effectively improve psoriatic lesions, atherosclerotic symptoms are also ameliorated in patients with both psoriasis and atherosclerosis. Also, IL-17A levels are highly expressed in the psoriatic lesions and atherosclerotic plaques. These clinical observations implicit that IL-17A could be a crucial link for psoriasis and atherosclerosis and IL-17A-induced inflammatory responses are the major contribution to the pathogenesis of comorbid psoriasis and atherosclerosis. In this review, the current literature related to epidemiology, genetic predisposition, and inflammatory mechanisms of comorbidity of psoriasis and atherosclerosis is summarized. We focus on the immunopathological effects of IL-17A in both diseases. The goal of this review is to provide the theoretical base for future preventing or treating psoriasis patients with atherosclerosis comorbidity. The current evidence support the notion that treatments targeting IL-17 seem to be hold some promise to reduce cardiovascular risk in patients with psoriasis.

## 1 Introduction

Psoriasis is a T cell-mediated inflammatory systemic disease mainly affecting the skin (1), which is characterized by a proliferation of keratinocytes and the appearance of erythematous plaques on the skin covered with white silver scales (2–4). Actually, psoriasis is not limited to the skin (5–7), and it also coexists with psoriatic arthritis, diabetes, depression, cardiovascular disease (CVD) and other diseases (8, 9). Among these diseases, CVD is the main cause of death in patients with psoriasis (9, 10). Comorbidity refers to the presence of one or more additional disorders that co-occur with the primary disease (11). Atherosclerosis, as a chronic inflammatory disease of large and medium-sized arteries, is a major cause of CVD (12). Patients suffering from comorbid psoriasis and atherosclerosis have brought a heavy burden to public health and health care system worldwide (5, 8). Although the mechanisms linking psoriasis with atherosclerosis are not well understood, it appears that over-activation of innate and adaptive immune responses is clearly involved in the two diseases (2, 13). Clinical research shows that the treatment of psoriasis with IL-17A inhibitors also reduces cardiovascular risk in patients with psoriasis (14, 15). Whether IL-17 is the pivotal factor for comorbid psoriasis and atherosclerosis needs to be clarified. Here we summarize the recent literature concerning the potential mechanisms of IL-17A that is involved in the comorbidity of psoriasis and atherosclerosis, thus providing a theoretical basis for IL-17A inhibitors treating patients with psoriasis and comorbid atherosclerotic disorders.

## 2 Epidemiology

### 2.1 Epidemiological Evidence in Comorbid Psoriasis and Atherosclerosis

Cumulative evidence shows that patients with psoriasis have a higher prevalence of cardiovascular events than healthy individuals at the same age (5). Dyslipidaemia is known to be one of the main risk factors for atherosclerosis (16). Epidemiological studies suggest a significant association between psoriasis and dyslipidaemia, with an odds ratio (OR) ranging from 1.04 to 5.55 (17–19). Patients with psoriasis had significantly lower levels of plasma high-density lipoprotein (HDL), and higher levels of total cholesterol, low-density lipoprotein (LDL), cholesterol, and triglycerides (TG) (20, 21), are similar to atherosclerosis. The patients with severe psoriasis are more likely to develop dyslipidaemia than patients with mild psoriasis (17). In addition, the prevalence of CVD was positively correlated with the severity of psoriatic lesions (5, 22). Patients with severe psoriasis had a 6.2% increased absolute risk of 10-year cardiovascular events compared to the age-matched health controls (23). If psoriasis was included to the Framingham cardiovascular risk score, most patients at low or moderate cardiovascular risk would be reclassified as being at moderate or high cardiovascular risk (24). Thus, psoriasis has been considered an independent risk factor for atherosclerosis, along with other conventional risk factors, such as smoking, hyperlipidaemia, diabetes, and hypertension (19, 25, 26).

### 2.2 Common Risk Factors in Comorbid Psoriasis and Atherosclerosis

Epidemiological studies have revealed that psoriasis and atherosclerosis share common external risk factors, which include smoking, alcohol abuse, sleep deprivation, environmental pollution, high-fat diets and psychological pressures (27, 28). Pathophysiologically, these risk factors activate the sympatho-adrenergic system, prolong an up-regulated reaction of the hypothalamic-pituitary-adrenal (HPA) axis, and promote endothelial dysfunction and lipid metabolism disorders (28, 29). These risk factors lead to systemically chronic inflammation that eventually contributes to development of both psoriasis and atherosclerosis (27–29).

Population-based prospective observational studies and randomized controlled trials also indicate some inherent risk factors that are common for both psoriasis and atherosclerotic disorders, such as hypertension, obesity, hyperlipidaemia and diabetes (28, 30, 31), of which obesity has more impact than other risk factors, because it also elevates the risk of diabetes (32, 33), hypertension (33–35), or dyslipidaemia (36, 37). Thus, obesity, a chronic metabolic disease, is the main factor in the association between psoriasis and CVD (38). The excessive accumulation of fat and abnormal fat distribution in the body is the consequence of inflammation and insulin resistance (39). Obesity is often accompanied by high levels of total cholesterol, TG, LDL, ultra-low-density lipoprotein (ULDL), and lipoproteins, as well as decreased levels of HDL and apolipoprotein B (ApoB) (40). In addition, adipokines are highly expressed in obese patients such as adiponectin, omentin, leptin, resistin, visfatin, retinol binding protein 4 (RBP4) and other chemo-attractants (41–43). All the adipokines, except adiponectin and omentin, appear to be involved in the pathogenic process of comorbid psoriasis and atherosclerosis by activating Th17 cells to secrete IL-17 (42–45).

## 3 Common Pathogenesis of Psoriasis and Atherosclerosis

### 3.1 Genetic Susceptibility

The common pathogenic mechanisms linking psoriasis and atherosclerosis are possibly driven by specific hub genes (46). Lu et al. noted the genetic similarity between psoriasis and CVD using single-nucleotide polymorphisms (SNPs) as risk indicators for CVD and psoriasis (46). Using the cyto-Hubba method, studies have recently identified 16 hub genes associated with these two diseases, in which 7 genes (*MMP9, CSF2RB, IL1RN, CCL5, CD53, NCF2* and *TLR2*) are upregulated in both psoriasis and atherosclerosis (47). Among them, the function of *MMP9* gene is more closely related to IL-17 as IL-17 inhibitors can reduce the expression of *MMP9* (48), and level of *MMP9* has been also associated with chronic progress of psoriasis (49, 50). Therefore, the pathogenic mechanisms of comorbid psoriasis and atherosclerosis are likely controlled by these hub genes (47). Animal models with gene knout out may be able to verify such comorbidity mechanisms at a genetic perspective.

### 3.2 Inflammatory Mechanism

#### 3.2.1 IL-17 Serves as a Key Mediator in Comorbid Psoriasis and Atherosclerosis

##### 3.2.1.1 The IL-17 Cytokine Family

**Figure 1:**
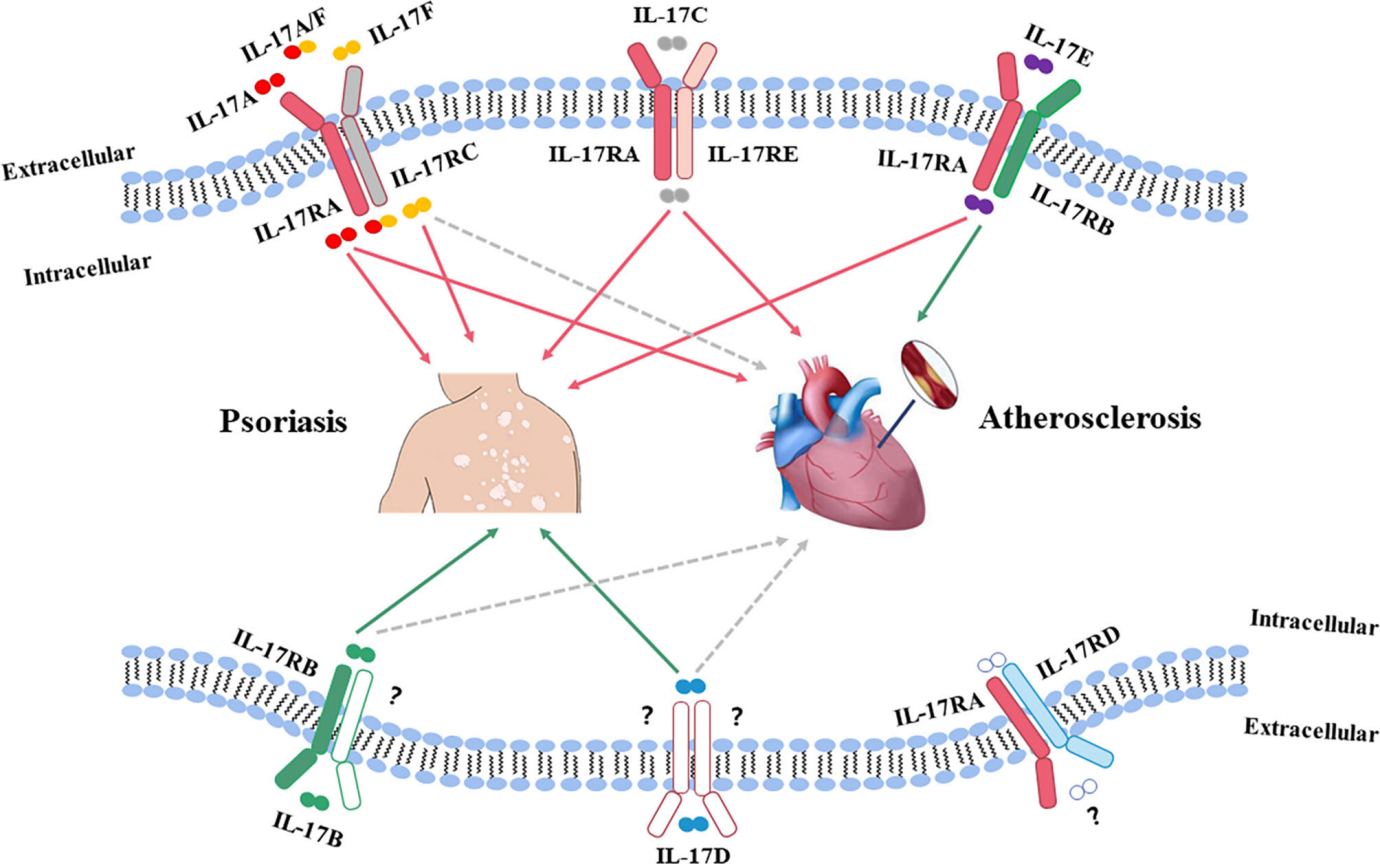
The roles of IL-17 in both psoriasis and atherosclerosis. The IL-17 family has six members (IL-17A to IL-17F), of which, IL-17A has the highest biological activity. Psoriatic lesions are characterized by high expression levels of IL-17A and IL-17F, which are believed to be responsible for tissue inflammation by recruiting neutrophils and forming micro abscesses on the sites. IL-17F acts in a manner similar to IL-17A in psoriatic lesions. Both IL-17A and IL-17C can aggravate psoriatic lesions and promote instability of atherosclerotic plaques. IL-17D has the highest homology with IL-17B, and the transcript levels of IL-17B and IL-17D are reduced in psoriatic lesions. However, the effects of IL-17B and IL-17D in atherosclerosis are still unknown. IL-17E expression is upregulated in psoriatic lesions, while IL-17E is protective against atherosclerosis. Red solid line: pathogenic role; Green solid line: non-pathogenic role; Gray dotted line: unknown function.

IL-17 is a main pro-inflammatory cytokine mainly produced by Th17 cells, which plays a crucial role in the pathogenesis of various inflammatory diseases, including psoriasis and atherosclerosis (51). The IL-17 cytokine family has six members (IL-17A to IL-17F) (52), of which IL-17A has the highest biological activity (53). Psoriatic lesions are characterized by high expression levels of IL-17A and IL-17F, which are responsible for tissue inflammation by recruiting neutrophils and forming micro-abscesses on the sites (54–56). In addition to IL-17A (57) and IL-17F (58), IL-17C (59, 60) and IL-17E (61, 62) can also aggravate psoriatic lesions; meanwhile, IL-17A and IL-17C can enhance atherosclerotic plaque instability, and IL-17E is protective against atherosclerosis (63, 64). However, the role of IL-17F in CVD has not yet been well investigated. The transcript levels of other two IL-17 subtypes, IL-17B and IL-17D, are reduced in psoriatic lesions (65), but the effects of IL-17B and IL-17D in atherosclerosis are unknown. These data indicate that the high levels of IL-17A and IL-17C may be more important in terms of disease development and progress in psoriatic lesions and atherosclerotic plaque. **Figure 1** shows the roles of IL-17 in both psoriasis and atherosclerosis (66). Heterodimeric receptors consisting of different members of the IL-17R family (IL-17RA to IL-17RE) activate the IL-17 signaling pathway upon binding to IL-17 (66). All subunits of the IL-17R family have a widespread expression pattern, with IL-17RA being ubiquitous (53, 66).

It is well known that IL-17 family members, especially IL-17A, play a critical role in psoriasis by activating other relevant cells to be involved in inflammatory response. It has been demonstrated that IL-17A can induce the proliferation of keratinocytes (67). In addition, it also targets other cell types, including endothelial cells, fibroblasts, chondrocytes and synovial cells, which may explain the IL-17A involvement in CVD (66, 68, 69). Thus, our discussion will focus on the role of IL-17A in comorbid psoriasis and atherosclerosis.

IL-17A is largely derived from Th17 cells that reside in lymphoid organs at a state of homeostasis but move into peripheral tissues in course of inflammation (70, 71). Peripheral organs, particularly mucous membranes and skin tissues, are the second major source of Th17 cells (70). Differentiation of Th17 cells requires the key transcription factor ROR-γt (retinoid-related orphan receptor) (72) in combination with other transcription factors, such as ROR-α, signal transducers, and transcriptional activators 3 (STAT3) (73, 74). At the same time, Th17 cells also highly express IL-23R for binding of IL-23, which amplifies Th17 cell proliferation and survival resulting in further IL-17 production (75). A microenvironment composed of transforming growth factor (TGF)-β and inflammatory cytokines IL-6, IL-21, IL-1β and IL-23 has been considered to play a central role in the proliferation and maintenance of Th17 cells as well (76). For example, highly expressed RORγt in γδ T cells, together with IL-23R and C-C chemokine receptor type 6 (CCR6), can significantly promote the production of IL-17A (77, 78).

In addition to Th17 cells, other immune cell types (e.g., γδ T cells and mast cells) can also produce IL-17A that accounts for a small proportion of IL-17A production. Two types of immune cells are responsible for IL-17A production: one is thymus-dependent cells type that includes CD8 T cells, γδ T cells and invariant NKT (iNKT) cells, and the other type is thymus-independent cells, including group 3 innate lymphoid cells (ILC), mast cells, and neutrophils (66, 79).

##### 3.2.1.2 IL-17 Is a Key Link in the Pathogenesis of Comorbid Psoriasis and Atherosclerosis

Studies using psoriasis-like animal models induced by air-pouch (80) or imiquimod (81) confirmed that psoriasis was elicited by the IL-23/IL-17 axis. Naive T cells are differentiated into Th1 cells by IL-12 and interferon (IFN)-γ or Th17 cells by IL-6, TGF-β and IL-23 respectively (82, 83). In psoriatic lesions, the activated Th1 cells produce large amount of tumor necrosis factor-alpha (TNF-α) and IFN-γ, which contribute to activation and proliferation of keratinocytes and subsequent expression of intercellular adhesion molecule 1 (ICAM-1) (84). ICAM-1 is bound to T cells to accelerate the migration of T cells, including Th17 subsets, into the dermis that gradually aggravates inflammation in psoriatic lesions (85). Increased ROR-γt expression and decreased Foxp3 expression in IL-17-expressing T cells then stimulate pro-inflammatory signaling, ultimately resulting in the activation and proliferation of keratinocytes in the skin (86). Activated Th17 cells secrete IL-22 and IL-17, which primarily promote the proliferation of keratinocytes to exacerbate inflammatory lesions. It is quite possible that IL-17 synergizes with TNF-α to promote the angiogenesis that is present in the psoriatic lesions (85, 87, 88).

**Figure 2:**
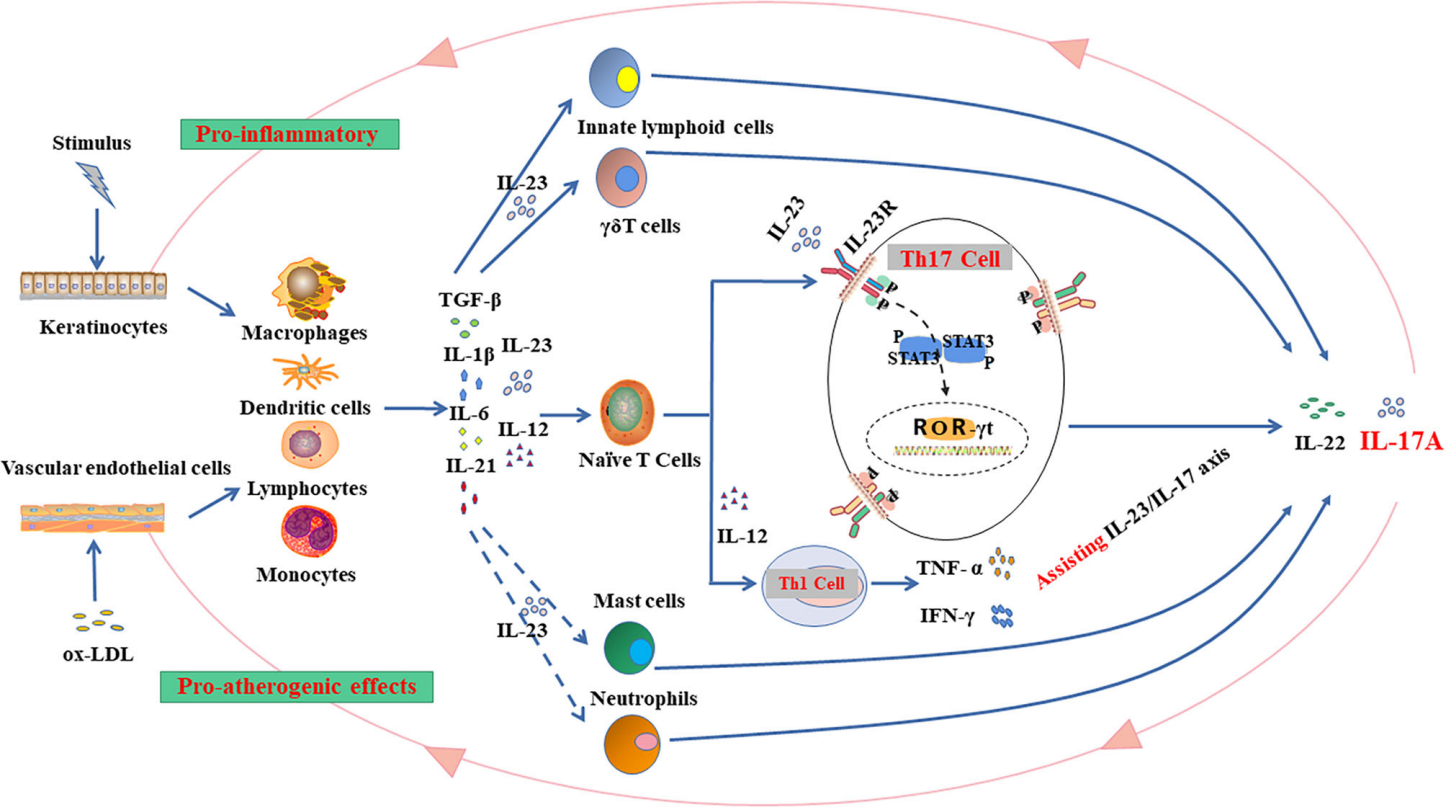
The IL-23/IL-17 axis in inflammation: Hypothetical mechanisms linking comorbid psoriasis and atherosclerosis. The shared molecular mechanisms, such as the IL-23/IL-17 inflammatory pathway, may exist in the pathogenesis of psoriasis and atherosclerosis. Stimulated keratinocytes and vascular endothelial cells will transfer signals to inflammatory cells (e.g., macrophages, dendritic cells, lymphocytes and monocytes) in skin epidermis or endothelium to release transforming growth factor (TGF)-β and inflammatory cytokines (e.g., IL-6, IL-21, IL-1β, IL-12, and IL-23). Thus, the inflammatory microenvironment emerges to stimulate the production of IL-17. IL-17 family includes 6 subtypes (IL17A-F), and the expressions of IL-17A and IL-17F are significantly elevated in the disease process. There are several types of IL-17-derived cells (e.g., innate lymphoid cells (ILCs), γ δ T cells, naïve T cells, mast cells, and neutrophils) and the main source of IL-17 is the differentiation of naive T cells into Th17 cells. Naive T cells can also be differentiated into Th1 cells that promote the production of IFN- γ and TNF-α to assist the IL-23/IL-17 axis in the pathogenesis of the diseases. Th17 cells also highly express IL-23R for IL-23 binding, which sustains IL-17 production and increases Th17 cell proliferation and survival. Th17 cell differentiation requires retinoid-related orphan receptor (ROR)-γt that cooperates with other transcription factors, including ROR-α, signal transducers and transcriptional activators 3 (STAT3) for resultant IL-17 production, which could further aggravate the inflammation on the epidermis and show proatherogenic effects on vascular endothelium. Current evidence tends to support the view that IL-17 exerts mainly pro-inflammatory role in the comorbid psoriasis and atherosclerosis.

Compared with psoriasis, the pathogenic effects of Th17 cells in atherosclerosis may rely more on the cytokines in microenvironment. Many previous studies have shown that nuclear factor (NF-kB) signaling pathway could be responsible for such proatherogenic effects of IL-17 (89, 90). Taleb et al. found that the dual production of IL-17 and IFN-γ would promote the atherosclerotic plaques instability, which could be also resulted from a decreased Treg cells by altered levels of TGF-β (91, 92). Actually, TGF-β3, drives a pathogenic Th17 phenotype by inducing IL-17A/IFN-γ double producing cells in an IL-23-dependent manner (93, 94). Dual production of IL-17 and IFN-γ will contribute significantly to the development of atherosclerotic plaques and increase plaque instability (91). Other studies found that increased level of circulating Th17 cells and Th17-associated cytokines is correlated with the severity and progression of carotid artery plaques in the presence of high IFN- γ milieu (95). In addition, IFN-γ, the landmark cytokine of Th1 cells, also plays an important role in atherosclerosis (96). Raymond et al. showed that IL-17, together with the IL-12/IFN-γ axis (Th1 cells related), can be detected in coronary artery-infiltrating T cells of human atherosclerotic plaques, suggesting infiltrating T cells can produce both IL-17 and IFN-γ. Nevertheless, IL-17 and IFN-γ exert synergistic pro-inflammatory rather than antagonistic effects, which can be observed in vascular smooth muscle cells in *in vitro* experiments (96–98). IL-17 can induce the expression of matrix metalloproteinase (MMP) and proinflammatory cytokines such as TNF-α, IL-6, granulocyte-macrophage colony-stimulating factor (GM-CSF), chemokines (CCL2, CXCL1, CXCL8, and CXCL10) in endothelial cells, smooth muscle cells, and macrophages to recruit neutrophils and monocytes into the plaques (99, 100). In atherosclerosis, endothelial cell activation at sites of nascent arterial plaque promotes monocyte and lymphocyte infiltration and subsequent activates macrophage and dendritic cell to secrete IL-12 and IL-23. Differentiated Th1 cells further promote atherosclerotic plaque growth, while Th17 cells enhance intraplaque neoangiogenesis and intraplaque hemorrhage (101). In addition, increased level of intraplaque IL-17 may further weaken the fibrous caps followed by plaque rupture (5, 92) (**Figure 2**).

Taken together, current evidence suggests that psoriasis and atherosclerosis may share common immunopathogenic mechanisms *via* the IL-23/IL-17 axis (18, 51). However, some studies also show that IL-17 plays a role in promoting the stabilization of atherosclerotic plaques (99, 100). It is believed that IL-17 maintains plaque stability by down-regulating VCAM-1 expression on endothelial cells and preventing T-cell infiltration into plaques, which in turn reduces inflammatory cytokines such as IFN-γ secretion and increases anti-inflammatory cytokines IL-5, IL-10, and TGF-β levels (18). Taleb et al. also proved that increased IL-10-producing Treg cells probably promote plaque stability (91). Increased levels of Treg cells promote conversion to TGF-β1, which is a TGF-β isoform in the generation of non-pathogenic Th17 cells (18). While the combination of TGF-β1 and IL-6 promotes the differentiation of Treg cells, the subsequent IL-10 provides anti-inflammatory and immune regulatory properties (102). The role of IL-17 in promoting either plaque instability or stability in atherosclerosis mainly depends on the inflammatory microenvironment that consists of Treg cells and cytokines. Current evidence tends to support the view that IL-17 exerts mainly pro-inflammatory role in the comorbid psoriasis and atherosclerosis.

#### 3.2.2 IL-17 Effects on Psoriasis and Atherosclerosis Can Be Enhanced by IFN-γ and TNF-α

The link between psoriasis and atherosclerosis has been proposed as “one syndrome and two plaques” (103, 104) to reflect the similar inflammatory cytokine profiles of the two diseases, including TNF-α, IFN-γ, IL-1, IL-6, IL-8, IL-12, IL-17, IL-20 and IL-23 (105), of which a synergistic effect of IFN-γ and TNF-α on the immunopathogenic mechanism has been noted in comorbid atherosclerosis and psoriasis (97).

Studies have shown that both IFN-γ and TNF-α cytokines are increased in the serum of patients with psoriasis and their respective receptors are increased in atherosclerosis (97). Elevated levels of IFN-γ and TNF-α, increased T-cell chelators and adhesion molecules, and progressive loss of endothelial barrier integrity are observed during atherosclerosis formation, both *in vivo* and *in vitro* experiments (97). Thus, the dual role of TNF-α/IFN-γ, especially TNF-α, also contributes significantly to psoriatic lesion inflammation and atherosclerosis formation (97). TNF-α, together with IL-17 in the IL-23/IL-17 axis at the core of pathogenesis, exerts a proinflammatory effect by promoting the maturation of myeloid dendritic cells (106, 107).

#### 3.2.3 Other cytokines and Chemokines Involved in Function of IL-17

In addition to TNF-α/IFN-γ and IL-23, many other chemokines and cytokines may contribute to the inflammatory process in comorbidity. It is worth noting that vascular endothelial growth factor (VEGF), IL-12 and monocyte chemotaxis protein 1 (MCP-1) are all strongly involved in the pathogenesis and development of both psoriasis and atherosclerosis (47). IL-17 could interact with its target cells such as keratinocytes and endothelial cells in a feedback loop manner (108). In the pathogenesis of psoriasis, IL-17 interacts with keratinocytes to promote the production of antimicrobial peptides, proinflammatory cytokines and chemokines (IL-1β, TNF-α, IL-6, IL-17C, CXCL1, CXCL3, CXCL5, CXCL8 [IL-8] and CCL20) and proliferative cytokines (IL-19) (55, 109, 110). IL-17 may promote endothelial inflammation *via* upregulating cytokines such as IL-6, IL-8 and ICAM-1, which in turn further promotes IL-17 production in a feedback loop for acceleration of the atherosclerotic plaque formation (109).

## 4 Pathophysiological Changes in Psoriasis and Atherosclerosis

Pathophysiologically, atherosclerosis and psoriasis share some common aspects. The infiltration of T cells, macrophages, monocytes, lymphocytes and dendritic cells are demonstrated in the psoriatic lesions and atherosclerotic plaques (105). In both diseases, inflammatory conditions could be an immune response for the locally formed autoantigens. These autoantigens stimulate both the innate and adaptive immune system for a T cell response (111).

As the key linker, IL-17 directly or indirectly mediates multiple steps of the immune signaling cascades (112, 113). In psoriatic inflammation, IL-17 drives the secretion of several key proinflammatory cytokines such as IL-1β, CCL20 and antimicrobial peptides, which can attract neutrophils, dendritic cells and T cells into psoriatic lesions (114). Histologically, the typical features of human psoriasis include hyperkeratosis, acanthosis, and infiltration of Th17 cells and neutrophils. In mouse psoriasis models, IL-17A released from skin-infiltrating T cells has been demonstrated to increase neutrophil recruitment (115, 116). An IL-17A dominance has also been shown in human psoriasis cases (117). Atherosclerosis is a complex inflammatory disease of the arterial wall that could be initiated by a variety of pro-atherogenic stimuli such as ox-LDL (118), and even represents autoimmune response. Microenvironment consisting of the aforementioned cytokines and chemokines is formed in the arterial endothelium after ox-LDL stimulation, in which monocytes and smooth muscle cells are infiltrated as atherosclerosis progresses (119). Increased blood flow and adhesion molecules induce migration of inflammatory cells into the vessel wall (2, 120). As a result, T cells-mediated adaptive immune response increases the number of arterial foam cells derived from macrophages and monocytes (121).

## 5 Treatments

Anti-inflammatory therapies directly or indirectly targeting IL-17A in patients with psoriasis are already available (122). IL-17 inhibitors are widely used in the treatment of psoriasis, especially in patients with moderate-to-severe psoriasis (122). IL-17A inhibitors can significantly improve the psoriatic lesions and pruritic symptoms of psoriasis patients (123). Upstream inflammatory factor inhibitors, such as TNF-α antagonists, significantly reduce the risk of CVD in psoriasis patients by reducing vascular inflammation, endothelial dysfunction, and arterial hypertension (97, 124). Some randomized clinical trials also revealed that secukinumab, a fully human monoclonal antibody against IL-17A, has a beneficial effect on CVD risk by promoting the endothelial function of plaque stability in psoriasis patients without CVD (15) and exhibits a neutral impact on aortic vascular inflammation and biomarkers of CVD (125). Clinically, the cardiovascular side effect of IL-17 inhibitors appears to be minimal, and IL-17 monoclonal antibodies improve outcomes of patients with psoriasis comorbid CVD by preventing lesion progression and sustaining plaque stabilization (126). In the same fashion, neutralization of cytokines downstream of IL-17A has been demonstrated to improve vascular health in a murine IL-17A over-expressed model (124). Anti-IL-17A monoclonal antibodies can prevent vascular disease in a mouse model of psoriasis, which will be the subject of future research focus (126). Other observational studies also support those systemic anti-inflammatory treatments may represent alternative approaches for the treatment of patients with comorbid psoriasis and atherosclerosis (127).

## 6 Conclusion

Current evidence from immunopathological studies has suggested that IL-17 and the IL-23/IL-17 axis may play a pivotal role in linking the comorbid psoriasis and atherosclerosis. Indeed, clinical trials have confirmed a decreased risk of early CVD indicators in psoriasis patients treated with IL-17 inhibitors. Thus, biological agents targeting IL-17A signaling pathway are expected to be widely used in the treatment of two comorbid diseases. While IL-17 promotes the proliferation of keratinocytes in psoriatic inflammatory lesions, its role in stabilizing or promoting plaque in atherosclerosis is still controversial. Current studies tend to support the pro-inflammatory pathogenic role of IL-17 in the comorbid psoriasis and atherosclerosis. A further characterization of different T cell subsets such as Treg cells in the course of both inflammatory diseases would provide new insights for future development of novel treatments.

However, it is worth to note that environmental pollution and poor lifestyle habits like smoking, alcohol abuse, sleep deprivation and psychological pressures are common external risk factors for both psoriasis and atherosclerosis. However, there is no literature indicating that poor lifestyle habits and adverse environments are linked with psoriasis and atherosclerosis through IL-17, which deserves further exploration. Clinically, patients with psoriasis, especially plaque psoriasis, who also suffer from the underlying diseases such as obesity, hyperlipidemia, diabetes, hypertension, and mental illnesses (e.g., anxiety and depression) should be routinely recommended for further assessments of cardiovascular risk.

## 7 Future Clinical Perspectives

Clinically, it has been proved that IL-17 inhibitors significantly improve the severity of psoriatic skin lesions and comorbid atherosclerotic plaques in psoriasis patients. The scientific community may pay more attention to the “plaque parallelism” of comorbid psoriasis and atherosclerosis, especially in plaque psoriasis. In view of the possible central role of IL-17 in the inflammatory pathway, future studies could shift the focus from IL-17 to its upstream and downstream related molecules, such as IL-6, to explore potential diagnostic biomarkers and targets of therapies for cardiovascular events in psoriasis patients.

## Author Contributions

DS and ZY: conception of the work. YW, CL and DS: extensive literature search and manuscript drafting. YW, JZ and CL: creating the cartoons. DS, ZY and YW: critical revision of the work and final version approval. All authors contributed to the article and approved the submitted version.

## Funding

This work was supported in part by grants from the National Natural Science Foundation of China (NM 81773337), the Key Research and Development Plan of Shandong Province (NM2019GSF108191) and the Traditional Chinese Medicine Science and Technology Program of Shandong Province (NM 2021M080).

## Conflict of Interest

The authors declare that the research was conducted in the absence of any commercial or financial relationships that could be construed as a potential conflict of interest.

## Publisher’s Note

All claims expressed in this article are solely those of the authors and do not necessarily represent those of their affiliated organizations, or those of the publisher, the editors and the reviewers. Any product that may be evaluated in this article, or claim that may be made by its manufacturer, is not guaranteed or endorsed by the publisher.
